# Signs of ROS-Associated Autophagy in Testis and Sperm in a Rat Model of Varicocele

**DOI:** 10.1155/2020/5140383

**Published:** 2020-04-13

**Authors:** Niloofar Sadeghi, Naeem Erfani-Majd, Marziyeh Tavalaee, Mohammad R. Tabandeh, Joël R. Drevet, Mohammad H. Nasr-Esfahani

**Affiliations:** ^1^Department of Reproductive Biotechnology, Reproductive Biomedicine Research Center, Royan Institute for Biotechnology, ACECR, Isfahan, Iran; ^2^Department of Histology, Faculty of Veterinary Medicine, Shahid Chamran University of Ahvaz, Ahvaz, Iran; ^3^Stem Cells and Transgenic Technology Research Center, Shahid Chamran University of Ahvaz, Ahvaz, Iran; ^4^Department of Basic Sciences, Division of Biochemistry and Molecular Biology, Faculty of Veterinary Medicine, Shahid Chamran University of Ahvaz, Ahvaz, Iran; ^5^GReD laboratory, CNRS UMR6293-INSERM U1103-Université Clermont Auvergne, Faculty of Medicine, CRBC Building, Clermont-Ferrand, France

## Abstract

Since autophagy was suspected to occur in the pathological situation of varicocele (VCL), we have attempted to confirm it here using a surgical model of varicocele-induced rats. Thirty Wistar rats were divided into three groups (varicocele/sham/control) and analyzed two months after the induction of varicocele. Testicular tissue sections and epididymal mature sperm were then monitored for classic features of varicocele, including disturbance of spermatogenesis, impaired testicular carbohydrate and lipid homeostasis, decreased sperm count, increased sperm nuclear immaturity and DNA damage, oxidative stress, and lipid peroxidation. At the same time, we evaluated the Atg7 protein content and LC3-II/LC3-1 protein ratio in testis and mature sperm cells, two typical markers of early and late cellular autophagy, respectively. We report here that testis and mature sperm show higher signs of autophagy in the varicocele group than in the control and sham groups, probably to try to mitigate the consequences of VCL on the testis and germ cells.

## 1. Introduction

Autophagy is considered to be a process conserved during evolution that plays an important role in physiological and pathological conditions. Its main role is the degradation of harmful cytoplasmic components such as damaged organelles and poorly folded proteins that are no longer needed. Thus, autophagy contributes to reduce the risk of formation of toxic protein aggregates [1] and promotes cell survival [2, 3]. This catabolic process can be activated under various stress conditions such as oxidative stress [4], thermal stress [5, endoplasmic reticulum stress [6], hypoxia [7], and unbalanced diet [8]. In pathological situations including infection and cancer and neurodegenerative, cardiovascular, and autoimmune diseases, the roles of autophagy have been well demonstrated [9, 10–12]. At the cellular level, during autophagy, some of the cytoplasmic proteins and organelles are sequestered into double membrane vesicular formations called “autophagosomes” that fuse with the lysosomes to degrade their contents. The resulting simple molecules, including free fatty acids, amino acids, and nucleotides, are then recycled and reused as an energy source by the cell [13].

According to recent reports, under physiological conditions, autophagy might contribute to spermatogenesis. In the mouse, the knockout of the autophagic gene Atg7 demonstrated its involvement in acrosome biogenesis by regulating the transport and/or fusion of proacrosomal vesicles derived from Golgi [12]. In human, it has been shown that autophagy is crucial for maintaining seminiferous tubules in stressful situations, such as exposure to formaldehyde [14]. Recently, research on male infertility has highlighted the pro-survival role of autophagy in the process of differentiating spermatogonia into spermatozoa [12].

Varicocele is probably one of the most controversial topics in the field of male infertility. Most infertility specialists worldwide doubt the etiology of varicocele or the effect of varicocelectomy on the treatment of male infertility [15]. Varicocele is described as the dilation and tortuosity of the sperm vein pampiniform plexus that leads to pathological problems affecting especially the left testicle [16]. The pathogenesis of varicocele-related infertility is not fully defined, although there are many hypotheses brought forward such as scrotal hyperthermia, oxidative stress, hypoxia, hormonal disorders, testicular hypoperfusion, and reflux of toxic metabolites [17]. In varicocele, a reflux of warm blood into the internal spermatic vein affects the testicular temperature exchange system. Thus, the resulting increase in testicular temperature of about 2.5°C and the testis inability to adjust the scrotal temperature disrupt spermatogenesis. This testicular hyperthermia is a source of oxidative stress inducing germ cell apoptosis and sperm DNA fragmentation as well as hormonal imbalance [18]. In addition, venous blood stasis in the dilated pampiniform plexus impedes arterial blood flow and reduces the supply of oxygen to testicular tissue, leading to testicular hypoxia [19].

Since oxidative stress, heat stress, and hypoxia are the main factors involved not only in the pathophysiology of varicocele [20] but also in the induction of autophagy [21, 22], we have sought to understand the relationship between autophagy and varicocele. The aim of this study was to create an experimental model of varicocele in rats to highlight the role of autophagy in VCL testicular tissue and sperm.

## 2. Materials and Methods

### 2.1. Study Population and Design

This study was approved by the Royan Institute's Institutional Review Board under code number 97000110. The animals were obtained from the Royan Institute for Biotechnology (Esfahan, Iran). All experiments were conducted according to the guidelines of the Royan Institute's Laboratory Animal Research Ethics Committee. Thirty Wistar rats (aged 4 weeks and weighing 150 to 200 grams) were maintained and housed in a controlled environment (12 hours of light and 12 hours of darkness at 24°C) with free access to standard food and water. The rats were randomly divided into 3 groups of 10 individuals. In the first group, the left varicocele was surgically induced (varicocele group) according to the protocol described in Ko et al. [23]. In the second group, the rats underwent a sham laparotomy (sham group). The third group consists of untreated rats (control group).

### 2.2. Surgical Technique and Outcome Assessment

Each rat was anaesthetized by intraperitoneal injection of a mixture of ketamine and xylazine. Then, the left renal vein was exposed via a median abdominal incision. In order to reduce the diameter of the renal vein to 1 mm, a 4.0 mm silk suture was performed around the left renal vein within the adrenal and sperm veins. This occlusion resulted in an increase in lateral intravenous pressure. This type of surgery led to the induction of a varicocele. After two months, all the rats were sacrificed, and their genitalia dissected. Morphometric parameters such as length, width, thickness, and weight of the left testicles were evaluated. After the dissected testicles were washed, a part of each testicle was fixed with Bouin for histological analysis. The remaining testicular tissues were used for the evaluation of the respective Atg7 and LC3 protein markers upstream and downstream of the autophagy pathway [21]. Left epididymides have also been dissected, and each epididymis has been divided into its three distinct segments: caput, corpus, and cauda [24]. For sperm retrieval, cauda epididymides were placed in a petri dish containing 5 ml of sperm washing medium (VitaSperm, Inoclon). Semen parameters, chromatin integrity, lipid peroxidation, and sperm Atg7 and LC3 protein contents were evaluated.

### 2.3. Assessment of Sperm Parameters

Sperm concentration and mobility were assessed using a sperm counting chamber (Sperm Meter; Sperm Processor, Aurangabad, India) and light microscopy. Eosin/nigrosine staining was used to assess sperm morphology. Briefly, the epididymal sperm were washed in PBS, then 30 *μ*l washed sperm were mixed with 60 *μ*l eosin stain (Merck, Darmstadt, Germany) for 3 minutes. In the next step, 90 *μ*l of nigrosine (Merck) was added to this mixture. For each sample, two smears were prepared, and 200 sperm were counted under an optical microscope. Abnormalities in the head, neck, and tail of sperm were determined, and a percentage of abnormal sperm morphology was reported for each sample [25].

### 2.4. Histomorphometric and Histochemical Studies

For histological studies, samples of testicular tissue fixed in Bouin were used. Paraffin blocks were prepared at 5-6 *μ*m and then stained with hematoxylin and eosin (HE), Schiff's periodic acid (PAS). Sudan Black staining in optimal cutting temperature compound (OCT) frozen sections was also carried out. These cryosections were cut using a cryostat (SLEE, Germany). For morphometric analysis, the Dino Lite digital lens and Dino Capture 2 software were used. Means of the spermiogenesis index (SI) and the tubal differential index (TDI) were considered for the evaluation of spermatogenesis. For the TDI index, the ratio of seminiferous tubules with four or more lines of differentiated cells of type A spermatogonia was calculated, while the ratio of seminal tubules containing spermatids was calculated for the SI index [26].

### 2.5. Evaluation of Sperm DNA Damage and Lipid Peroxidation

The assessment of DNA damage and lipid peroxidation in epididymal sperm was performed using the orange acridine dye (Merck) and the BODIPY w 581/591 C11 assay (D3861, Molecular Probes), respectively, as previously in Afiyani et al. [25] and Aitken et al. [27]. The results of these two tests were expressed as “percentage of DNA fragmentation” and “lipid peroxidation.”.

### 2.6. Antioxidant Activity Assessment in Testis

For the evaluation of antioxidant capacities of testicular tissue, the total protein concentration was determined by the Biuret method (Pars Azmoon kit, Iran) and using the BT-1500 automatic analyzer. Catalase activity (CAT) was measured at 37°C by following the rate of disappearance of H_2_O_2_ at 240 nm [28]. The activities of glutathione peroxidase (GPX) and superoxide dismutase (SOD) were measured using commercial kits, respectively, from (ZellBio GmbH, Germany) for GPX and (Ransod-Randox Lab, Antrim, UK) for SOD. In addition, the level of malondialdehyde (MDA), an important marker of lipid peroxidation, was calculated in homogenized tissues by detecting the absorption of reactive substances of thiobarbituric acid at 532 nm [29].

### 2.7. Assessment of Atg7 and LC3 Autophagic Proteins by Immunohistochemistry

Paraffin-embedded Boin's fixed testis 5-6 *μ*m sections were prepared for the evaluation of the proteins Atg7, LC3-I, and LC3-II. Briefly, the sections were dewaxed, dehydrated, and blocked with blocking serum, then incubated overnight at 4°C with primary antibodies [rabbit antibodies anti-Atg7/LC3 (1 : 1000, Abcam)] and [anti-*β*-actin (1 : 1000, Sigma-Aldrich)]. Then, the slides were washed three times with PBS containing 0.05% Tween-20. Subsequently, the slides were incubated with a secondary antibody [IgG-FITC goat rabbit (Sigma, 1 : 1000)] for 1 hour at 37°C. The slides were then washed and observed under an Olympus fluorescent microscope (BX51) equipped with a HBO100 mercury vapor lamp stabilized at 490 nm excitation.

### 2.8. Western Blotting

We evaluated the expression of Atg7, LC3-I, and LC3-II by Western blot according to the modified protocol of Foroozan-Broojeni et al. [30]. In short, epididymal sperm samples and testicular tissues were washed with PBS, and protein extraction was performed with the TRI reagent (Sigma-Aldrich, USA). Protein concentrations were determined using the Bradford method (Bio-Rad, USA). For each sample, 30 *μ*g of protein was mixed in the loading buffer and heated at 100°C for 5 minutes. Electrophoresis was performed on a 12% SDS polyacrylamide gel; then, the proteins were transferred to polyvinylidene fluoride (PVDF) membranes (Bio-Rad, USA). The membranes were blocked with PBS containing 10% skimmed milk powder (Merck, USA). For the detection of Atg7, LC3, and control (*β*-actin) proteins, we used, respectively, a rabbit anti-Atg7 polyclonal antibody from Abcam (Cambridge, MA, USA) at 1 : 1000 dilution, a Novus Biologicals rabbit anti-LC3 polyclonal antibodies (Littleton, CO, USA) at 1 : 4000 dilution as specific primary antibodies, and an anti-*β*-actin antibody (Sigma-Aldrich) at 1 : 1000 dilution. For the first two antibodies, the membranes were exposed overnight while for the *β*-actin antibody, the membrane was exposed for 90 minutes. The membranes were then washed three times and incubated for one hour with secondary antibodies. For anti-Atg7 and anti-LC3 antibodies, the secondary antibody was a rabbit antirabbit IgG conjugated to horseradish peroxidase (HRP) (Dako, Japan) while for *β*-actin antibody, it was a goat antimouse IgG conjugated to horseradish peroxidase (HRP) (Dako, Japan). The membranes were then washed three times. The presence of specific proteins was identified using a Western Blot ECL Advance detection kit (Amersham, GE Healthcare, Germany). For data quantification, the densities of the protein bands were analyzed using the 1-D Quantity One v 4.6.9 analysis software (Bio-Rad, Munchen, Germany). Data normalization was calculated by dividing the densities of the Atg7 and LC3 bands by the density of the *β*-actin band and represented as the state of expression of Atg7 and LC3, respectively. In addition, the ratio between LC3-II and LC3-I was measured as an indicator of autophagic level. It should be noted that, given the similar results for the above-mentioned parameters between the control and simulated groups, we decided to compare only the autophagic proteins of the control group with those of the varicocele group.

#### 2.8.1. Statistical Analysis

For data analysis, we used Statistical Package for the Social Sciences for Windows, version 18.0. Shapiro-Wilk and Levene tests were performed for normal distribution and equal variance, respectively. All the results of this study were presented as a mean ± standard deviation (SD). To compare the data between the three groups, the one-way analysis of variance (ANOVA) followed by the Tukey HSD test was used. The differences were considered statistically significant when *P* < 0.05.

## 3. Results

### 3.1. Classic Testicular VCL Alterations Are Accompanied by an Autophagic Response

**Figure 1:**
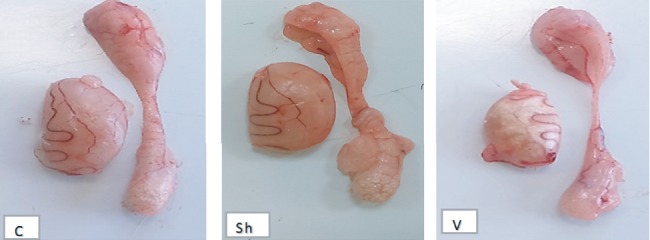
Representative photographs of the macroscopic appearance of the testes and epididymis in the control (C), sham (Sh), and varicocele (V) groups.

**Figure 2:**
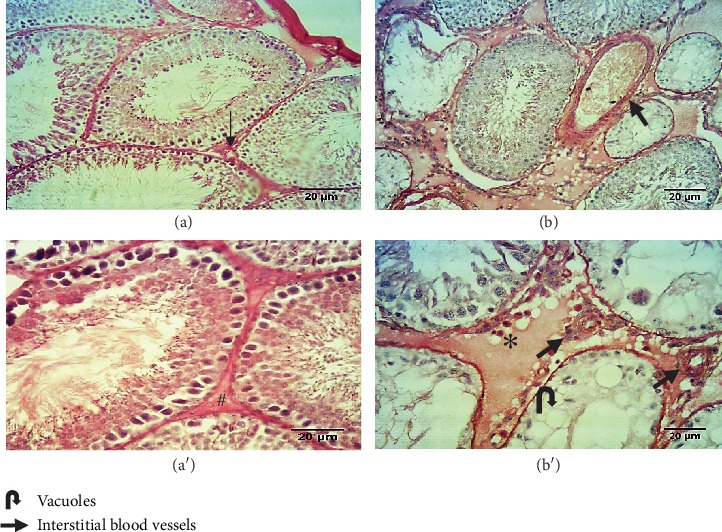
(a and a') HE sections of the testes from the control group showing the normal structure of the seminiferous tubules with normal interstitial tissue (#). Note the few interstitial blood vessels (bold **⟶**). (b and b') HE sections of the testis from the varicocele group. The germ cells appear disorganized with vacuoles and intercellular gaps. Note the numerous blood vessels (black bold arrows) in the interstitial connective tissue with edema (∗) (magnification, X100 and X200).

In this study, the length, width, thickness, weight, and volume of the testes in each group were measured and found significantly reduced when the control group was compared with the VCL group for all the parameters (mean testis length: 1.61 ± 0.07*vs*1.46 ± 0.12; *P* < 0.001; width: 0.86 ± 0.08*vs*0.74 ± 0.07; *P* < 0.001; thickness: 0.60 ± 0.08*vs*0.38 ± 0.06; *P* < 0.001; and volume: 1.46 ± 0.15*vs*1.1 ± 0.30; *P* < 0.001) with the exception of the testis weight (see Figure 1). No significant difference was found for any of the monitored parameters when the control group was compared to the sham operated one. At the histological level, the induction of varicocele promoted degenerative changes in the seminiferous tubules and an increase in edema and blood vessels in the testicular interstitial tissue. Sperm cells were not seen in the lumen of some of the seminiferous tubules, indicating that spermatogenesis arrests occurred. Such phenomena were not observed in the testis of the control group (see Figure 2). As shown in Table 1, TDI and SI were significantly lower in the varicocele group than in the control group (*P* < 0.001).

**Figure 3:**
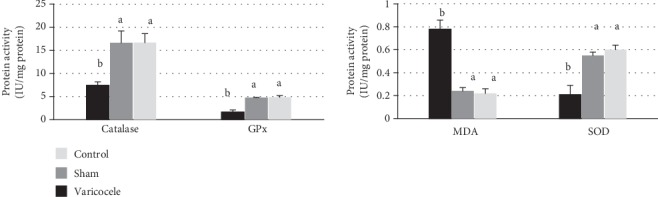
Graphs showing the evaluation of the AO activities of the SOD/CAT/GPx catalytic triad and the level of lipid peroxidation (MDA) between the VCL, sham, and control groups. Different letters indicate significant differences between groups at *P* < 0.05. GPx: glutathione peroxidase; CAT: catalase; SOD: superoxide dismutase; MDA: malondialdehyde.

Since VCL is known to be associated with testicular oxidative stress, we monitored the enzymatic activities of the major testicular primary antioxidants, namely, CAT, GPX, and SOD, in the 3 groups. As shown in Figure 3, the mean CAT activity decreased to 7.46 ± 1.23 in the VCL group from 16.67 ± 3.36 and 16.64 ± 3.61 (*P* < 0.05), respectively, in the control and sham groups. Identically, the GPX activity decreased to 1.69 ± 0.72 in the VCL group while it was 4.86 ± 0.64 and 4.8 ± 0.05 (*P* < 0.05) in the control and sham groups, respectively. Finally, SOD activity also decreased to 0.21 ± 0.03 while it was 0.6 ± 0.11 and 0.55 ± 0.05 (*P* < 0.05) in the control and sham groups, respectively. Thus, all major antioxidant enzyme activities were significantly lower in the varicocele group than in the control and sham groups. In addition, the mean testicular MDA content was significantly (*P* < 0.05) higher in the VCL group (0.78 ± 0.15) than in the control (0.22 ± 0.08) and sham (0.24 ± 0.04) groups, and no differences were observed between the control and sham groups.

**Figure 4:**
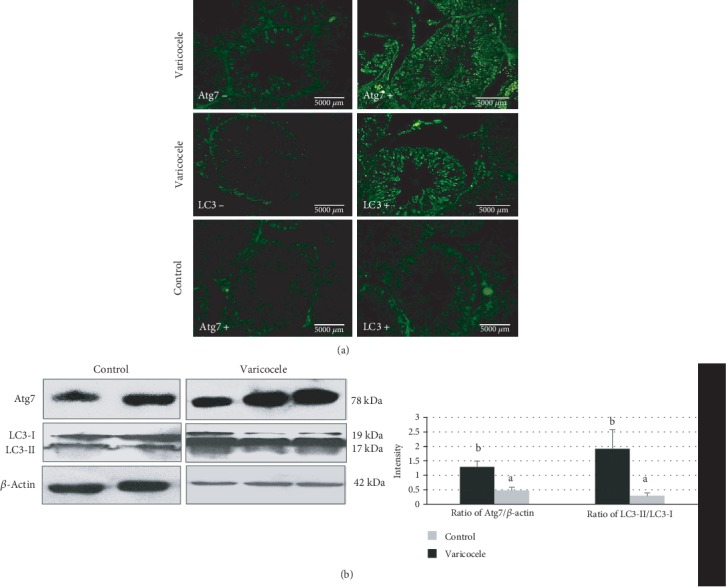
(a) Comparative immunohistochemical analysis of Atg7 and LC3 abundance in paraffin sections prepared from rat testes between the varicocele and control groups (scale bar 5000 *μ*m). (b) Representative Western blots illustrating the protein content in Atg7, LC3-I, and LC3-II in the left testicles of the control and varicocele groups (left panel). The intensity of the Atg7 protein band has been normalized with *β*-actin and the LC3-II/LC3-I ratio are presented in the bar graphs (right panel). Different letters indicate significant differences between groups at *P* < 0.05.

To evaluate whether autophagic processes were ongoing in the VCL testis, the testicular autophagic protein content of Atg7 and LC3, respectively, as early and late markers of autophagy, were monitored by immunohistochemistry and Western blot. As shown in Figure 4(a), Atg7 and LC3 protein signals were higher in cells of the left testis (VCL testis) near the seminiferous tubule basal membrane and lumen corresponding to spermatogonia, elongated spermatids, and spermatozoa in the VCL group compared to the control group.  Figure 4(b) shows the Atg7, LC3-I, and LC3-II protein contents as evaluated by Western blot. Atg7 expression was significantly higher in the VCL group than in the control group (respectively, 1.29 ± 0.44*vs*0.48 ± 0.21; *P* = 0.013). In addition, the LC3-II/LC3-I ratio was also significantly higher in the VCL group than in the control group (respectively, 1.92 ± 1.31*vs*0.29 ± 0.24; *P* = 0.016).

### 3.2. VCL Testis Shows Altered Carbohydrates and Lipid Contents

**Figure 5:**
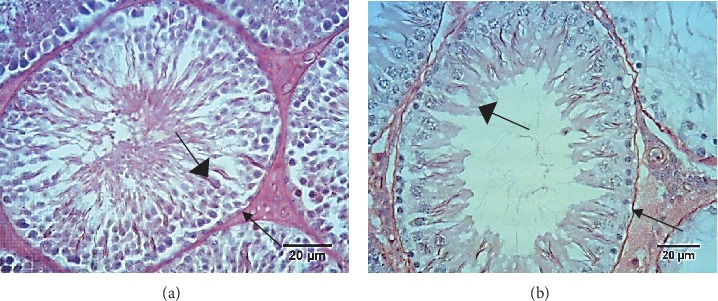
Cross-section of seminiferous tubules. (a) Control group: most cells have an intense PAS response (bold arrows). The narrow arrow (⟶) points to the thin regular basal membrane of the seminiferous tubules. (b) Varicocele group: the majority of cells have a poor response to PAS (bold arrows). The narrow arrow (⟶) points to the thickened basal membrane of the seminiferous tubule (magnification, X200).

**Figure 6:**
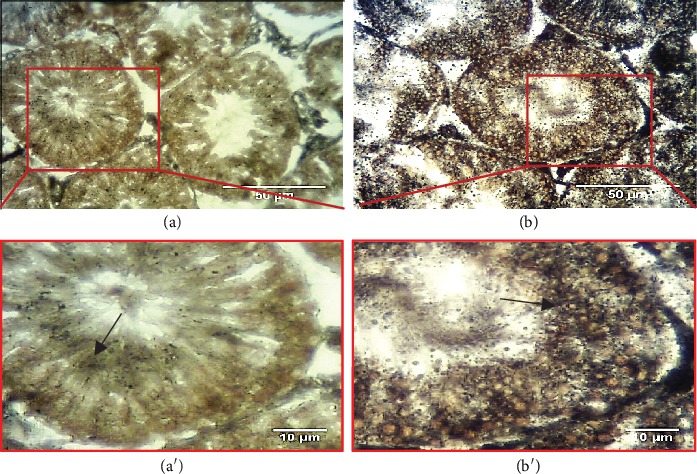
Frozen sections of seminiferous tubules. (a) Control group: most of the cells are presenting faint SB reaction (⟶); (a') red square is enlarged. (b) Varicocele group: high amounts of lipids were seen in germ cells (⟶); (b') red square is enlarged (SB, magnification, X400).

As shown in Figure 5, staining of testicular tissue sections using PAS showed that the control germ cells had a cytoplasm highly reactive to PAS. In addition, the control seminiferous tubules were surrounded by a thin regular basal membrane. In contrast, in the VCL group, spermatogonia and spermatocytes appeared to have a lower PAS reactivity and a thickened basement membrane. As shown in Figure 6, we also evaluated the accumulation of lipids in the germ cell cytoplasm by SB staining. We observed an increase in the cellular lipid content in the VCL group compared to the control group as evidenced by the observation that the majority of spermatogonia showed dense lipid foci in their cytoplasm.

### 3.3. VCL Sperm Cells Also Show Signs of Autophagy

As classically expected in VCL situation, the mean sperm concentration was 110.2 ± 6.28, 100.3 ± 9.49, and 68.8 ± 10.84 in the control, sham, and varicocele groups, respectively, attesting of a significant reduction in the VCL group compared to the control (*P* < 0.001) and sham (*P* = 0.005) groups. The mean percentage of sperm mobility was 90.2 ± 3.7, 78.8 ± 5.51, and 46.0 ± 5.03 in the control, sham, and VCL groups, respectively, showing a significant reduction in the VCL group compared to the control (*P* < 0.001) and sham (*P* = 0.04) groups. In addition, the mean percentage of abnormal sperm morphology was 6.4 ± 1.62, 8.2 ± 1.33, and 9.8 ± 1.07 in the control, sham, and VCL groups, respectively, showing a significant increase in the VCL group compared to the control (*P* < 0.001) and sham (*P* = 0.012) groups (see Table 2).

In addition, and again as expected, VCL sperm presented classical alterations. Sperm DNA damage as evaluated by acridine orange staining was significantly higher (*P* < 0.05) in the VCL group than in the control and sham groups (see Table 2). The evaluation of sperm lipid peroxidation by BODIPY C11 showed that the percentage of positive BODIPY C11 sperm cells was significantly higher (*P* < 0.05) in the VCL group than in the control and sham groups (Table 2). No difference was observed between the control group and the sham group for the two parameters evaluated.

**Figure 7:**
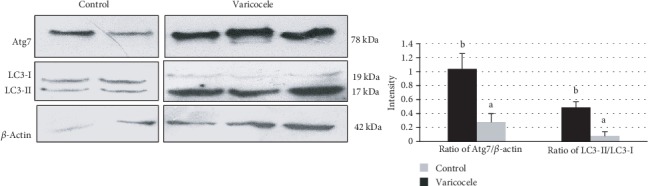
Expression level of the proteins Atg7, LC3-I, and LC3-II in the sperm of the control and varicocele groups evaluated by Western blot. The Atg7 protein level (normalized by *β*-actin) and the LC3-II/LC3-I ratio are presented in the bar graph (right panel). Different letters indicate significant differences between groups at *P* < 0.05.

As for the testicular tissue, we monitored the autophagic Atg7 protein content in spermatozoa and found that it was significantly higher (*P* < 0.05) in the VCL group than in the control group. In addition, the sperm LC3-II/LC3-I ratio was also found significantly higher (*P* < 0.05) in the VCL group than in the control group (see Figure 7).

## 4. Discussion

In accordance with earlier studies [18, 31], we show here that the induction of VCL in rat results in reduction of testis weight and testicular volume that have been related to loss of germ cells by apoptosis because of the highly sensitive nature of spermatogenesis to hyperthermia [27]. Indeed, previous reports showed that varicocele activates apoptosis in seminiferous epithelial cells leading to Sertoli-mediated phagocytosis of apoptotic germ cells [21, 22, 32, 33]. The induction of VCL in the present rat model was also accompanied by testicular histopathological defects including reduced germinal epithelium, increased basement membrane thickness, increased interstitial blood vessels, and acidophilic material leading to spermatogenesis disruption as well as poor sperm count and quality as described elsewhere [22, 24, 34].

The literature suggests that in the hypoxic state, aerobic glycolysis is impaired and that activation of anaerobic glycolysis for lactate and pyruvate production and the induction of lipolysis should take place, which is not the case in the varicocele situation although it is associated with hypoxia. In support of this observation, Razi et al. [24] suggested that any disorder in glucose and hexose carbohydrate transport and/or metabolism could drive the testis cells to switch from glucose to lipids as main source of energy. In VCL, however, due to the impaired blood circulation and its associated situation of glucose deficiency (the main source of anaerobic glycolysis), neither lactate and pyruvate are accumulated, nor cells switch to lipolysis; therefore, this is most likely why lipids droplets are observed. On the other hand, it should be noted that the intracellular lipid content of the Sertoli cell largely depends on its phagocytosis activities of residual bodies and/or apoptotic sperm cells. Thus, intracytoplasmic lipid foci in the first layers may be the result of such activities [33]. Moreover, it was shown that there is in VCL a limited rate of gluconeogenesis in the testis [35]. In agreement with these characteristics and as reported already in Razi and Malekinejad [33], Abdel-Dayem [36], and Bayomy et al. [37], we also observed in our VCL animal group a decreased testicular cell cytoplasmic carbohydrate content via PAS staining. This observation was also associated with increased lipid accumulation in the cytoplasm of VCL seminiferous tubule cells.

It was proposed that although these new pathways for energy supply have been shown to help to support testicular cell needs in VCL testis, they are not sufficient, in fine leading to autophagy cell starvation [33]. Because of low glucose supply, germ cells were reported to face a decrease in the pentose phosphate-pathway (PPP) flux and, consequently, in the NADPH/NADP ratio that is so important for the GSH-dependent antioxidant (AO) systems [35]. This resulted in a decrease of the cell antioxidant capacity [18, 38, 39]. In addition, testicular hyperthermia associated with VCL also contributes to shift the AO/ROS balance towards oxidative stress [20, 40]. We are here in total agreement with the literature since we have observed that in the VCL group, all the major primary AO activities (SOD, CAT & GPX) were reduced and that the tissue MDA content was significantly increased signing a clear state of oxidative stress. Altogether, these data suggest that in the present VCL-induced rat model, we have all the classical features associated with VCL including heat stress, hypoxia and oxidative stress, and disturbed carbohydrate/lipid homeostasis [21, 22]. These conditions were recently suspected to activate autophagy as a pro-survival testicular cell response [12] providing on the one hand a way to eliminate damaged proteins and organelles as could result from cellular oxidative stress and, on the other hand, a way to fuel testicular cells with alternative sources of nutrients in order to maintain cell homeostasis [11]. This is line with the pro-survival and protective role of autophagy as was seen elsewhere in situation of starvation [41, 42].

Using two markers of the cell autophagic response pathway, the upstream Atg7 protein and the downstream LC3-II/LC3-I protein ratio, we show here that in the VCL testis and also in mature spermatozoa, both markers were significantly more present when compared to the sham or/and control situations. This in agreement with Zhang et al. [21] who have shown that autophagy was induced in mouse male germ cells after heat stress and that down-expression of Atg7 lowers this heat-stress-associated autophagic response. It also concurs with Zhu et al. [22] who recently reported that the HIF-1*α*/BNIP3/Beclin1 autophagy signaling pathway was upregulated in the VCL rat testis. These authors hypothesized that upon VCL, early hypoxia damages seminiferous cells, organelles, and proteins triggering the autophagic response as a pro-survival process. It is suspected that although autophagy is triggered as a pro-survival process, it does not succeed in protecting the testis, mainly because VCL is a permanent situation of stress that finally leads to apoptosis [22, 41, 43]. VCL-induced autophagy is not a surprising finding as oxidative stress, a well-known consequence of VCL, is known to promote mitophagy as a way to remove ROS-damaged mitochondria, the major source of additional intracellular ROS in situation of oxidative stress [44].

VCL-induced germ cell autophagy is also not a surprise as it was shown elsewhere that in cells showing severe DNA damage autophagy is triggered to delay the apoptotic response as a way to provide the energy needed for DNA repair [45]. The higher spermatozoa content in Atg7 and LC3-II/LC3-I ratio we noticed in the VCL animal group is likely to be a testimony of the germ cell autophagic response. These results are consistent with an earlier study reporting that after 2 hours of testicular heat stress (at 37°C), sperm cells showed a higher LC3-II/LC3-I ratio [46]. It also concurs with a very recent study showing that these two autophagic markers (Atg7 and LC3-II/LC3-I ratio) were found to be elevated in infertile VCL males [30]. In that same study, it was also observed a reduction in sperm concentration and motility as well as an increase in sperm DNA damage, abnormal morphology, and lipid peroxidation, all features we have encountered in our rat model.

## 5. Conclusions

**Figure 8:**
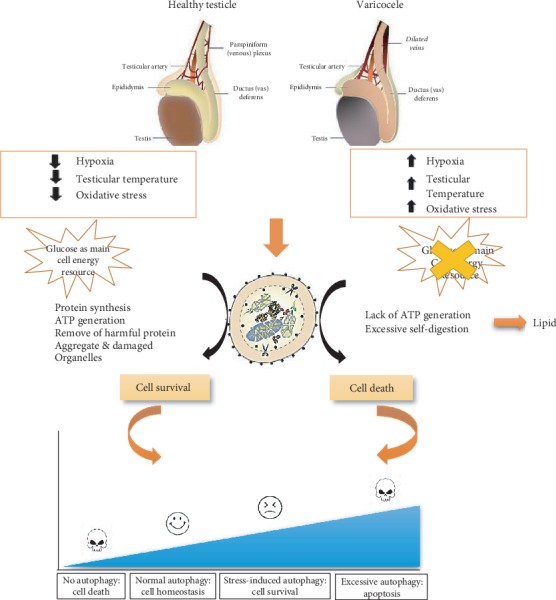
Schematic diagram summarizing the involvement of autophagic processes in normal healthy testis or VCL. VCL-induced cellular damage defines the level of autophagy that can be considered either as a pro-survival response or as a pro-death additive signal leading to apoptosis.

In conclusion, we confirm here that autophagy is triggered in the VCL testis. Whether autophagy is engaged prior to/or along with apoptosis has not been demonstrated yet and will have to wait a further detailed kinetic analysis. Logically, autophagy should precede apoptosis as it is classically a pro-survival pathway mitigating the apoptotic death pathway by providing alternative energy sources to sustain cell metabolism. However, some authors have suggested that a prolonged state of autophagy, as it is the case in the VCL context, may constitute an additional proapoptotic signal when lipolysis fails to efficiently replace glycolysis [21, 22, 32, 47]. We attempted to illustrate this in Figure 8.

## Figures and Tables

**Table 1:** Comparison of TDI and SI between control and varicocele groups.

Variable^c^	Control	Varicocele	*P* value
TDI (%)	94.21 ± 3.75^a^	47.13 ± 7.33^b^	<0.001
SI (%)	85.3 ± 2.8^a^	41.0 ± 5.3^b^	<0.001

Legend: Data are presented as means ± SD. Different letters indicate significant differences between groups at *P* < 0.05. TDI: tubular differential index; SI: spermiogenesis index.

**Table 2:** Comparison of sperm parameters, mean percentage sperm with DNA damage, and lipid peroxidation between control, sham, and varicocele groups.

Variable^c^	Control	Sham	Varicocele	*P* value
Concentration (10^6^/ml)	110.2 ± 6.28^a^	100.3 ± 9.49^a^	68.8 ± 10.84^b^	<0.001^∗^
				0.005^∗∗^

Motility (%)	90.2 ± 3.70^a^	78.8 ± 5.51^a^	46.0 ± 5.03^b^	<0.001^∗^
				0.04^∗∗^

Abnormal morphology (%)	6.4 ± 1.62^a^	8.2 ± 1.33^a^	9.8 ± 1.07^b^	<0.001^∗^
				0.012^∗∗^

%DNA damage	42.3 ± 20.33^a^	40.4 ± 10.45^a^	54.7 ± 17.77^b^	0.02^∗^
				0.03^∗∗^

%Lipid peroxidation	7.7 ± 3.62^a^	8 ± 4.3^a^	40.7 ± 4.13^b^	<0.001^∗^
				<0.001^∗∗^

Legend: Data are presented as means ± SD. Different letters indicate significant differences between groups at *P* < 0.05. ^∗^Varicocele to control. ^∗∗^Varicocele to sham.

## Data Availability

All data reported are presented in the manuscript.
